# Resistin Contribution to Cardiovascular Risk in Chronic Kidney Disease Male Patients

**DOI:** 10.3390/cells12070999

**Published:** 2023-03-24

**Authors:** Katarzyna Romejko, Aleksandra Rymarz, Katarzyna Szamotulska, Zbigniew Bartoszewicz, Tomasz Rozmyslowicz, Stanisław Niemczyk

**Affiliations:** 1 Department of Internal Diseases, Nephrology and Dialysis, Military Institute of Medicine-National Research Institute, 04-141 Warsaw, Poland; arymarz@wim.mil.pl (A.R.); sniemczyk@wim.mil.pl (S.N.); 2 Department of Epidemiology and Biostatistics, Institute of Mother and Child, 01-211 Warsaw, Poland; katarzyna.szamotulska@imid.med.pl; 3 Department of Internal Diseases and Endocrinology, Medical University of Warsaw, 02-097 Warsaw, Poland; zbigniew.bartoszewicz@wum.edu.pl; 4 Department of Pathology and Laboratory Medicine, Perelman School of Medicine, University of Pennsylvania, Philadelphia, PA 19104, USA; rozmyslo@pennmedicine.upenn.edu

**Keywords:** cardiovascular risk, chronic kidney disease, resistin, plasminogen activator inhibitor-1

## Abstract

Background: Resistin is a molecule that belongs to the Resistin-Like Molecules family (RELMs), the group of proteins taking part in inflammatory processes. Increased resistin concentrations are observed in cardiovascular complications. Resistin contributes to the onset of atherosclerosis and intensifies the atherosclerotic processes. The aim of this study was to investigate the relationship between resistin and cardiovascular (CV) risk in men with chronic kidney disease (CKD) not treated with dialysis. Materials and Methods: One hundred and forty-two men were included in the study: 99 men with eGFR lower than 60 mL/min/1.73 m^2^ and 43 men with eGFR ≥ 60 mL/min/1.73 m^2^. CV risk was assessed. Serum resistin, tumor necrosis factor-alpha (TNF-alpha) and plasminogen activator inhibitor-1 (PAI-1) were measured among other biochemical parameters. Results: We observed that resistin concentrations were significantly higher in patients with CKD compared to individuals with eGFR ≥ 60 mL/min/1.73 m^2^ (*p* = 0.003). In CKD, after estimating the general linear model (GLM), we found that resistin is associated with CV risk (*p* = 0.026) and PAI-1 serum concentrations (0.012). The relationship of PAI-1 with resistin depends on the level of CV risk in CKD (*p* = 0.048). Conclusions: Resistin concentrations rise with the increase of CV risk in CKD patients and thus resistin may contribute to the progression of cardiovascular risk in this group of patients. The relationship between resistin and CV risk is modified by PAI-1 concentrations.

## 1. Introduction

Resistin is a 12.5 kDa cysteine-rich polipeptide belonging to the Resistin-Like Molecules (RELMs) family, a group of proteins that initiate inflammatory processes [1]. Resistin in humans is mainly produced by macrophages, granulocytes, monocytes and bone marrow cells. It was also found in the hypothalamus, pituitary gland, thymus, skeletal muscle, digestive system, pancreas and placenta [2]. Resistin was initially defined as adipocytokine. However, the production of resistin in adipocytes depends on species with its intense synthesis in mice adipose tissue [3]. In humans, resistin is released mainly by inflammatory cells, thus acting as a biomarker of inflammation [4]. It is still not known whether a specific receptor for resistin exists. Toll-like receptor 4 (TLR4) and adenyl cyclase-associated protein-1 (CAP1) are probably functional receptors for resistin. The interaction of resistin with TLR4 and CAP1 results in the increase of serum inflammatory cytokine concentrations and vascular dysfunction [5,6]. Initially, resistin was thought to be related mainly with the development of insulin resistance [7]. It is nowadays known that resistin is highly expressed in numerous inflammatory diseases such as osteoarthritis, septic shock and acute pancreatitis, and it is also increased in autoimmune processes such as rheumatoid arthritis and lupus erythematosus [8,9,10,11]. Resistin levels are higher in obese individuals [12]. Its concentrations are positively associated with the value of waist-to-hip ratio and BMI. Patients with metabolic syndrome have higher resistin concentrations compared to healthy individuals [13]. Resistin concentrations are elevated in type 2 diabetes mellitus [14].

High resistin levels are also found in cardiovascular complications. Hypertensive patients have increased resistin concentrations compared to individuals with correct blood pressure [15]. There is growing evidence that resistin takes part in the onset and development of atherosclerosis. Resistin increases the accumulation of lipids in macrophages and thus takes part in the formation of foam cells [16]. Resistin stimulates endothelial dysfunction, downregulates vasorelaxation, enhances thrombosis, is involved in angiogenesis and the proliferation of vascular smooth muscle cells and increases cell adhesion [17,18,19,20]. Because resistin aggravates atherosclerosis in this mechanism, it is involved in the development of coronary and peripheral artery disease [21]. It has been found that resistin may also increase the risk of myocardial infarction [22]. After acute coronary syndrome, high resistin concentrations potentiate myocardial remodelling with increased myocardial fibrosis, the dilation of the left ventricle and the decreased left ventricle contractility [23]. Moreover, resistin may also enhance the risk of heart failure [24].

CKD is one of the fastest growing causes of death [25]. Cardiovascular complications are the main cause of morbidity and mortality in CKD [26]. With the decrease of eGFR, the risk of cardiovascular and all-cause mortality increases and is the highest in patients treated with dialysis [27]. High resistin concentrations in CKD are due to decreased GFR and, in consequence, low resistin elimination through the kidneys [4,28,29]. On the other hand, it has been proven that increased resistin concentrations are associated with higher risk of kidney function decline. The mechanism by which resistin may accelerate the deterioration in kidney function is not yet well known, but the probable reason is that resistin enhances the synthesis of pro-inflammatory cytokines and intensifies oxidative stress, which consequently induces glomeruli dysfunction [30]. Increased resistin levels in CKD may possibly play a role in the aggravation of subclinical inflammatory states observed in this group of patients. It has been recently suggested that resistin acting through the CAP1 receptor in CKD may increase pro-inflammatory processes and accelerate atherosclerosis in this group of patients [31]. It was also found that elevated serum resistin level is an independent predictor of cardiovascular and all-cause mortality in CKD [32]. Increased resistin concentrations in CKD may also contribute to the loss of protein resources and may be involved in the development of malnutrition-inflammation states and, as a consequence, protein energy wasting, which is nowadays thought to be strongly associated with poor survival in this group of patients [33,34].

Atherosclerotic cardiovascular disease (ASCVD) is the main cause of morbidity and mortality in the general population [35]. The estimation of cardiovascular (CV) risk is crucial for intervention on an individual level and to implementing the appropriate preventive or therapeutic procedures for the patient. The estimation of CV risk enables classifying patients into four CV risk categories: low-risk, moderate-risk, high-risk and very high-risk. The classification includes the value of eGFR, the presence of diabetes mellitus, significantly elevated risk factors, such as the concentration of total cholesterol > 310 mg/mL or blood pressure ≥ 180/110 mmHg and documented cardiovascular disease, shown to be clinical or unequivocal on imaging. It also requires calculating the 10-year risk of fatal CV disease based on the SCORE system. The SCORE chart is a CV risk assessment tool that helps to estimate the magnitude of CV risk according to sex, age, systolic blood pressure, total cholesterol concentration and smoking status [36].

The purpose of our study was to investigate the relationship between resistin and CV risk in men with chronic kidney disease not treated with dialysis.

## 2. Materials and Methods

### 2.1. Design

We performed an observational cross-sectional study in two groups of male patients—with CKD not treated with dialysis and individuals with eGFR ≥ 60 mL/min/1.73 m^2^.

### 2.2. Patients

The study samples consisted of two groups of men: 99 men with CKD and eGFR lower than 60 mL/min/1.73 m^2^ (study group) and 43 men with eGFR ≥ 60 mL/min/1.73 m^2^. We performed our research between November 2018 and February 2020. Patients with CKD who were recruited to the study visited the Nephrological Outpatient Clinic of the Military Institute of Medicine in Warsaw, Poland, for a routine check-up. Participants without CKD were recruited from the department of internal medicine. The inclusion criteria were age between 18 and 80 years and eGFR < 60 mL/min/1.73 m^2^ for the group of patients with CKD and eGFR ≥ 60 mL/min/1.73 m^2^ for participants without CKD. The exclusion criteria were the lack of consent to participate in the study, renal replacement therapy or its requirement within the following 3 months for patients with CKD, clinical signs of infection and the presence of metal parts in the body. Each participant signed an informed consent. The study protocol was accepted by the local ethics committee (Bioethics Committee in Military Institute of Medicine in Warsaw, Poland, IRB acceptance number 120/WIM/2018, obtained 22 August 2018).

Blood samples were taken after an overnight fast and were transported to the local Department of Laboratory Diagnostics. Serum creatinine concentrations were measured using the Jaffe method (Gen.2; Roche Diagnostics GmbH, Risch-Rotkreuz, Switzerland). Samples for measuring resistin, tumor necrosis factor-alpha (TNF-alpha) and plasminogen activator inhibitor-1 (PAI-1) levels were kept frozen at −80 °C. Resistin, TNF-alpha and PAI-1 concentrations were assessed using the Bio-Plex MAGPIX (Luminex Corporation, Austin, TX, USA).

eGFR was calculated according to the short Modification of Diet in Renal Disease (MDRD) formula: GFR in mL/min per 1.73 m^2^ = 175 × SerumCr − 1.154 × age − 0.203 × 1.212 (if patient is black) × 0.742 (if female).

### 2.3. Defining the Cardiovascular Risk

The CV risk was assessed based on the 2016 European Guidelines on cardiovascular disease prevention in clinical practice. Participants with CKD were classified as high- or very high-CV risk. Patients without CKD were classified into 4 CV risk groups: low-risk, moderate-risk, high-risk and very high-risk. Selected chronic diseases and elevated biochemical parameters were taken into account. Additionally, the 10-year risk of fatal cardiovascular disease was estimated based on the SCORE system specific for the country [36]. For cases with nonignorable missing data on smoking status (39 CKD patients and 4 patients with eGFR ≥ 60 mL/min/1.73 m^2^), the scores were calculated as the average value for smokers and non-smokers. A sensitivity analysis was performed in order to take into account the consequences of possible misclassification bias related to improper assumption regarding smoking status. Two different scenarios were considered: (1) in which all patients with missing data on smoking status were coded as non-smokers and (2) in which all patients with missing data on smoking status were coded as smokers (see Supplementary File).

### 2.4. Statistical Analysis

The results are presented as medians and interquartile ranges (IQR). The Kolmogorov–Smirnov test was used for evaluating distributions for normality. For correlation analysis, Spearman ρ was applied. Differences between groups were assessed using the non-parametric Mann–Whitney test. Hypotheses regarding gradual changes across categories were verified using Jonckheere–Terpstra test for trends. Uni- and multivariate analyses of relationships between continuous outcomes and continuous and categorical variables were performed by application of the General Linear Model (GLM) and analysis of residuals. A *p*-value < 0.05 was considered to be statistically significant. Statistical analysis was performed using IBM SPSS v. 25.0, Armonk, NY, USA: IBM Corp. 

## 3. Results

Patients with CKD were older compared to individuals with eGFR ≥ 60 mL/min/1.73 m^2^ (*p* = 0.005). The median age in CKD was 66 years and in patients without CKD 57 years. We found statistically significant differences in resistin concentrations between the two groups—resistin levels were significantly higher in CKD patients (*p* = 0.003). Moreover, resistin concentrations increased with the decrease of eGFR in both groups (ρ_s_ = −0.451, *p* < 0.001 in CKD patients and ρ_s_ = −0.345, *p* = 0.023 in individuals with eGFR ≥ 60 mL/min/1.73 m^2^). Inflammatory parameters such as TNF-alpha and CRP were also higher in patients with eGFR < 60 mL/min/1.73 m^2^ compared to those with eGFR ≥ 60 mL/min/1.73 m^2^ (*p* < 0.001, *p* = 0.020). The median value of serum PAI-1 concentrations was higher in participants without CKD than in those with impaired kidney function (*p* = 0.008). We also found statistically significant differences in total cholesterol, non-HDL cholesterol and triglyceride concentrations between the two groups. The median values of total cholesterol and non-HDL cholesterol were higher in participants with eGFR ≥ 60 mL/min/1.73 m^2^ compared to CKD patients (*p* = 0.014, *p* = 0.011). Triglyceride concentrations were significantly higher in the CKD group (*p* = 0.024). Patients with CKD had higher systolic and diastolic blood pressure compared to those with eGFR ≥ 60 mL/min/1.73 m^2^ (*p* = 0.006, *p*= 0.001) (Table 1).

We classified all participants according to CV risk. We found that 70 patients with CKD were very high-CV risk and 29 participants were high-CV risk. Among participants with eGFR ≥ 60 mL/min/1.73 m^2^, 3 patients were at low CV-risk, 14 individuals were at moderate-CV risk, 19 were categorized as being at high-CV risk and 7 participants were included in the group with very high-CV risk. The median values of resistin concentrations differed between the groups according to CV risk. In CKD, resistin concentrations were significantly higher in patients with very high-CV risk compared to those with high-CV risk (*p* = 0.014). In individuals with eGFR ≥ 60 mL/min/1.73 m^2^, we also observed that resistin concentrations rose with the increase of CV risk, with the highest resistin level in those with very high-CV risk and the lowest in patients with low-CV risk; however, the trend was at the border of significance (p_trend_ = 0.087). We did not find statistically significant differences in resistin concentrations between the study group and patients with eGFR ≥ 60 mL/min/1.73 m^2^ who were at the same CV risk: very high and high (*p* = 0.451, *p* = 0.736) (Table 2).

In the univariate general linear models (GLM) with resistin as a dependent variable and CV risk degree as an independent variable, CV risk degree explained only 5.3% of the resistin variation in the CKD group and 12.1% of the resistin variation in patients with eGFR ≥ 60 mL/min/1.73 m^2^.

To explore the unexplained variation of resistin, a correlation analysis between residuals from the above-mentioned GLMs and potential resistin-related variables not included in the CV risk estimation was used. TNF-alpha, CRP, PAI-1 concentrations and BMI were taken into account. In the study group, there was a statistically significant association between resistin residuals and TNF-alpha (ρ_s_ = 0.204, *p* = 0.043) and PAI-1 (ρ_s_ = 0.208, *p* = 0.038), but not with CRP and BMI. In participants with eGFR ≥ 60 mL/min/1.73 m^2^, there was a statistically significant association between resistin residuals and PAI-1 concentrations (ρ_s_ = 0.553, *p* < 0.001), but not with TNF-alpha, CRP and BMI.

When the above-mentioned statistically significant variables were added to the previously estimated GLMs models and interaction terms were taken into account, the explained variation of resistin concentrations in CKD patients increased from 5.3% to 14.9% and in participants with eGFR ≥ 60 mL/min/1.73 m^2^ from 12.1% to 45.7% (Table 3). In CKD patients, resistin concentration was higher in the case of very high-CV risk than in high-CV risk (*p* = 0.026) and increased with the elevation in PAI-1 concentrations (*p* = 0.012). However, the effect of PAI-1 concentrations was dependent on the level of CV risk (*p* = 0.048 for the interaction term). In the presence of CV risk and PAI-1 concentration variables, TNF-alpha was not statistically significant.

**Figure 1:**
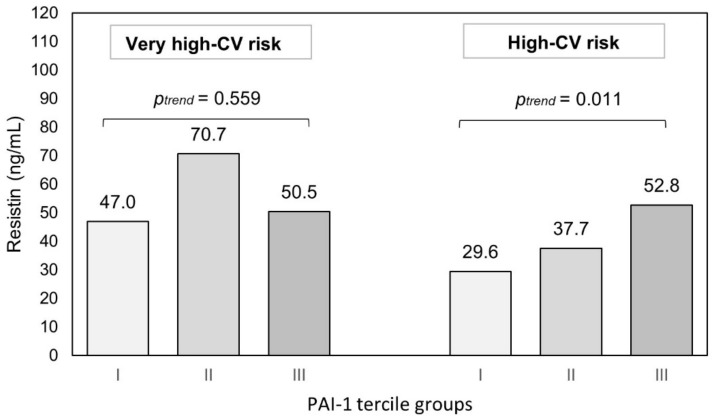
Resistin concentration and PAI-1 levels (tercile groups) in CKD patients by level of CV risk.

To illustrate the GLMs estimated relationships, a univariate stratified analysis was performed separately in the subgroups of CKD patients with very high risk and high risk of CV. For these analyses, PAI-1 values were categorized into tercile groups (Figure 1). Patients with CKD and very high-CV risk had the highest resistin concentrations. Resistin levels in participants with CKD and high-CV risk were lower compared to those with very high-CV risk and were associated with high PAI-1 levels. The concentrations of resistin in CKD patients and very high-CV risk was elevated independently of PAI-1 concentrations (p_trend_ = 0.559). In participants with CKD and high-CV risk, the concentrations of PAI-1 increased with the rise of resistin level (p_trend_ = 0.011). Median concentration of resistin in the highest tercile group of PAI-1 in the high-CV risk stratum was similar to median concentration of resistin in the very high-CV stratum taken as a whole.

In patients with eGFR ≥ 60 mL/min/1.73 m^2^, serum PAI-1 concentrations were associated with the level of resistin but due to the small group of participants without CKD, the estimation of variables was not justified. However, in patients with eGFR ≥ 60 mL/min/1.73 m^2^ and very high-, high- and moderate-CV risk, the PAI-1 concentrations rose with the increase of resistin level, regardless of CV risk. For patients with very high-CV risk, the Spearman correlation coefficient was 0.900 (*p* = 0.037) for individuals with high-CV risk—0.510 (*p* = 0.022) and for participants with moderate-CV risk—0.464 (*p* = 0.095). We did not test this relationship in the group of patients with low-CV risk due to small number of subjects (*n* = 3).

## 4. Discussion

In our study, we found that resistin concentrations rise with the increase of CV risk. Numerous recent data have reported that resistin is a molecule that is involved in cardiovascular complications. Resistin is nowadays known to take part in the onset and development of atherosclerosis. There are several mechanisms by which resistin participates in atherosclerotic processes. Resistin is synthesised by monocytes that are recruited from the blood to subendothelial spaces where they differentiate into macrophages. Macrophages accumulate atherogenic lipoproteins such as very-low-density lipoprotein (VLDL), intermediate-density lipoprotein (IDL), LDL and then become foam cells that play a crucial role in the onset of atherosclerotic plaque. Resistin increases the accumulation of lipids in macrophages and thus takes part in the formation of foam cells [16,37]. Resistin may also accelerate the development of atherosclerosis through its contribution to endothelial dysfunction. Resistin increases the release of endothelin-1 (ET-1), vascular cell adhesion molecule-1 (VCAM-1), intercellular adhesion molecule-1 (ICAM-1), vascular endothelial growth factor receptors (VEGFRs), matrix metalloproteinases (MMPs) and monocyte chemotactic protein-1 (MCP-1), which results in endothelial dysfunction and thus may promote the advancement of atherosclerosis [18,38,39]. Moreover, resistin induces pentraxin 3, an inflammatory cytokine involved in atherosclerotic processes in human endothelial cells [38]. Resistin also accelerates the expression of integrins via the P38/MAPK pathway [40]. Additionally, resistin inhibits the expression of tumor necrosis factor (TNF) receptor-associated factor-3 (TRAF-3), which acts as an inhibitor of TNF receptor superfamily member 5 (CD 40) [41]. A new study by Tisato found that resistin decreases the expression of paroxonase 1 (PON1), which is a protein with atheroprotective properties. By lowering the expression of PON1, resistin exerts its pro-inflammatory and pro-atherogenic functions [42]. An additional mechanism through which resistin exerts an pro-atherogenic effect is the increase of proprotein convertase subtilisin/kexin type 9 (PCSK9) cellular expression. PSCK9 is a protease that enhances intracellular LDL receptor (LDLR) degradation and, as a consequence, elevates serum LDL concentrations. It was reported that in obese individuals, resistin decreased LDLR expression up to 40% [43]. The concentrations of resistin are higher in unstable compared to stable atherosclerotic plaques [44]. Moreover, resistin increases the synthesis of apolipoprotein B and thus accelerates the process of atherosclerosis by inducing dyslipidemia with high LDL, triglycerides and low HDL concentrations [45].

In our study, we investigated whether there is an association between resistin and CV risk in a group of patients with CKD. We estimated cardiovascular risk based on the 2016 European Guidelines on cardiovascular disease prevention in clinical practice, which takes into account selected chronic diseases and elevated biochemical parameters as well as the 10-year risk of fatal cardiovascular disease estimated using the SCORE system. Since cardiovascular complications are the main cause of morbidity and mortality in CKD, it is crucial not only to prevent the decrease in eGFR, which in itself increases the cardiovascular risk, but also to implement preventive and therapeutic procedures that would enable downregulating the progress of cardiovascular complications in this group of patients. Resistin concentrations increase with the decline of kidney function, probably due to lower elimination of resistin by the kidneys [4,28,29]. There is growing evidence that increased resistin concentrations may also accelerate a decline of kidney function [30]. Resistin presumably enhances the synthesis of pro-inflammatory cytokines and oxidative stress, which consequently induce glomeruli dysfunction [30]. The study by Norman reported the negative impact of high resistin concentrations on eGFR, similar to the impact on systolic blood pressure, which was stronger than conventional risk factors such as metabolic syndrome [46]. In our study, we found that resistin concentrations are significantly higher in CKD compared to patients with eGFR ≥ 60 mL/min/1.73 m^2^ (*p* = 0.003). Moreover, resistin concentrations rise with the decrease of eGFR in both groups (*p* < 0.001 in CKD patients and *p* = 0.023 in participants with eGFR ≥ 60 mL/min/1.73 m^2^). We also found that resistin concentrations rise with the increase of cardiovascular risk in both groups—CKD and with eGFR ≥ 60 mL/min/1.73 m^2^. In CKD patients, resistin was significantly higher in the group with very high-CV risk compared to high-CV risk (*p* = 0.011). We also observed the rise of resistin concentrations in patients with eGFR ≥ 60 mL/min/1.73 m^2^ with the increase of CV risk, although the results were at the border of significance (p_trend_ = 0.087).

Increased inflammatory processes play a crucial role in the development of cardiovascular complications such as hypertension, hypercholesterolemia and coronary artery disease. Low-grade inflammation enhances oxidative stress [47,48]. The role of oxidative stress in atherosclerosis was for the first time described by Ohara in 1993 [49]. Chronic kidney disease is also associated with increased low-grade inflammatory status, which has an impact on cardiovascular complications in this group of patients. Moreover, high inflammatory cytokin level in the CKD population may lead to the loss of protein resources and, as a consequence, may also lead to to cachexia and sarcopenia, which are associated with a worse outcome [50]. Anti-inflammatory treatment in patients with CKD results in the reduction of major adverse cardiovascular events [51]. Inflammatory status in CKD is multifactorial [52]. It has been found that elevated resistin concentrations in CKD are also associated with an increased inflammatory state. In the study by Yaturu, resistin concentrations correlated with increased levels of TNF-alpha in CKD participants, which suggests that resistin may play a role in the development of sub-clinical inflammation in CKD [53]. Additionally, a positive relationship between resistin and other inflammatory markers such as C-reactive protein (CRP), interleukin-6 (IL-6) and TNF-alpha was reported [4,34]. In our study, we found that TNF-alpha concentrations rise with the increase of resistin levels, unexplained by CV risk in CKD (*p* = 0.043). The probable reason for why we did not report such a relationship in individuals without CKD is the small number of participants in this group.

Thrombosis plays a crucial role in the progression and complications of atherosclerosis. It has been proven that resistin also has procoagulatory functions. Resistin enhances the expression of tissue factor (TF) and thus promotes a prothrombotic state [13,17]. In in vitro studies, resistin increased the expression of apolipoprotein C-I, an angiotensin-converting enzyme, and TNF receptor superfamily member 1A and CD40, which suggests that resistin may possibly induce thrombotic complications by influencing lipoprotein metabolism and stimulating inflammation [13]. Additionally, in an animal model, resistin promoted thrombosis by up-regulating the expression of MMP-1 and MMP-9 [54]. There are numerous studies that have found that the procoagulatory effect of resistin occurs through the intensification of PAI-1 synthesis [13,54,55,56,57].

PAI-1 is one of the most crucial inhibitors of the plasminogen/plasmin system [58]. Plasminogen is a proenzyme that can be converted into plasmin, an active form of enzymes [59]. The role of plasmin is to degrade fibrin and thus prevent intravascular thrombosis. The conversion of plasminogen into plasmin takes place through the action of PAs (plasminogen activators) [59]. Plasmin production is also controlled by inhibitors that prevent excessive cloth lysis. PAI-1 is one of the strongest antifibrinolytic proteins that binds to the tissue-type plasminogen activator (tPA) or urokinase-type plasminogen activator (uPA), inhibiting their function and reducing plasmin production [60]. It is released from platelets, monocytes, the vascular endothelium, adipose tissue, liver, vascular smooth muscle cells and cardiac monocytes. PAI-1 is known to act as a proatherothrombotic factor that takes part in the development of atherosclerotic plaque and is also involved in cardiovascular complications, including coronary artery disease [61,62,63]. Elevated PAI-1 expression was found in severely atherosclerotic human arteries [64]. Myocardial infarction in most cases is caused by a plaque rupture with the exposure of a procoagulant top. This results in the formation of an occlusive thrombus. Increased PAI-1 levels in atherosclerotic lesions accelerate thrombus formation after the plaque rupture. The consequence of increased PAI-1 concentrations is also lower fibrinolytic activity and thus the inability to manage a thrombus [65]. PAI-1 concentrations rise with the decrease of eGFR [66].

In our study, serum concentrations of PAI-1 were significantly higher in patients with eGFR ≥ 60 mL/min/1.73 m^2^ compared to CKD. In our data, serum PAI-1 concentrations increased with the rise of resistin levels unexplained by CV risk in both groups—CKD (*p* = 0.038) and participants with eGFR ≥ 60 mL/min/1.73 m^2^ (*p* < 0.001). In the final GLM models, we found that the addition of PAI-1 to CV risk increases the explained variation of resistin from 5.3% to 14.9% in the CKD group and from 12.1% to 45.7% in individuals with eGFR ≥ 60 mL/min/1.73 m^2^. A significant and, in our opinion, promising result of our study is that resistin is associated with cardiovascular risk, both in those with eGFR < 60 mL/min/1.73 m^2^ and eGFR ≥ 60 mL/min/1.73 m^2^. Patients with CKD and high-CV risk had lower resistin concentrations than participants with CKD and very high-CV risk. In the group of patients with CKD and high-CV risk, increased resistin concentrations were associated with high PAI-1 levels. This relationship between resistin and PAI-1 was not observed in CKD patients with very high-CV risk, where high resistin levels were independent of PAI-1 concentrations. Resistin concentrations were also associated with PAI-1 in participants with eGFR ≥ 60 mL/min/1.73 m^2^. Although this group was small, we found that PAI-1 concentrations rise with the increase of resistin level independently of CV risk (*p* = 0.037 for patients with very high-CV risk, *p* = 0.022 for individuals with high-CV risk and *p* = 0.095 for participants with moderate-CV risk).

Some studies reported that obese individuals have higher serum resistin concentrations compared to those with correct body mass [12,13]. The study of Owecki, which included 136 obese subjects and 48 non-obese patients, proved a positive association between resistin and BMI—obese patients had higher resistin levels compared to healthy controls [67]. However, some studies did not show the relationship between obesity and resistin. The report of Hasegawa with 209 participants found no association between resistin and BMI [68]. Additionally, the study of Lee reported that resistin did not correlate with such markers of adiposity as BMI, waist-to-hip ratio and fat mass [69]. In our study, we did not find a relationship between residual serum resistin concentrations and BMI in both CKD patients and participants with eGFR ≥ 60 mL/min/1.73 m^2^.

Our report has some limitations. This is a cross-sectional study that was performed in two groups. The sample size is relatively small, especially in the group of patients with eGFR ≥ 60 mL/min/1.73 m^2^. Participants with eGFR ≥ 60 mL/min/1.73 m^2^ are younger than patients with CKD. The study with female participants would also enable the comparison of results according to gender. An additional limitation is that we did not have information on the smoking status of CKD patients; thus, we assumed the average number of points in the SCORE system assigned for smoking status. However, we also assessed the results if participants with nonignorable missing data on smoking status were either smokers or non-smokers. The results of this sensitivity analysis that are included in the Supplementary File do not conflict with the results presented in the main body of the manuscript. Moreover, our study was conducted before the new classification of cardiovascular disease risk was published—the 2021 ESC Guidelines on cardiovascular disease prevention in clinical practice; therefore, we relied on the 2016 European Guidelines on cardiovascular disease prevention in clinical practice. The 2016 guidelines do not include albumin-to-creatinine ratio stratification but take into account eGFR only. This may induce some measurement bias in the classification of CKD, which in our study seems to be negligible since our participants were observed in the Nephrological Outpatient Clinic for more than 3 months and were diagnosed and treated as CKD patients.

## 5. Conclusions

After conducting this study with CKD patients and participants without a kidney function decrease, we may conclude that resistin concentrations rise not only with the progression of kidney function decrease and with the fall of eGFR, but also with the increase of CV risk in CKD; thus, resistin may contribute to the progression of cardiovascular risk in this group of patients. The relationship between resistin and CV risk is modified by PAI-1 concentrations.

## Figures and Tables

**Table 1:** Clinical data of the studied samples.

	eGFR < 60 mL/min/1.73 m^2^ *n* = 99	eGFR ≥ 60 mL/min/1.73 m^2^ *n* = 43	*p*-Value
Age [years]	66.0 (59.0–71.0)	57.0 (41.0–70.0)	**0.005**
eGFR [mL/min/1.73 m^2^]	36.0 (23.0–46.0)	95.0 (76.0–110.0)	**<0.001**
Total cholesterol [mg/dL]	165.0 (143.0–207.0)	196.0 (163.0–221.0)	**0.014**
Low-density lipoprotein cholesterol [mg/dL]	103.0 (80.0–139.0)	119.5 (93.7–159.1)	0.084
High-density lipoprotein cholesterol [mg/dL]	42.0 (35.0–54.0)	46.1 (40.0–54.4)	0.170
Non-high-density lipoprotein cholesterol [mg/dL]	123.0 (93.0–166.0)	145.6 (116.5–173.9)	**0.011**
Triglycerides [mg/dL]	144.0 (108.0–224.0)	127.0 (93.0–161.0)	**0.024**
BMI [kg/m^2^]	28.6 (25.4–33.4)	28.4 (24.4–32.3)	0.374
HgbA1c ≥ 6.5 [%]	24.2%	11.9%	0.097
Serum glucose [mg/dL]	97.5 (86.5–132.5)	97.0 (89.0–103.0)	0.833
HOMA-IR	3.8 (1.9–7.9)	2.4 (1.5–6.8)	0.189
SBP [mmHg]	130.0 (125.0–140.0)	130.0 (113.8–135.0)	**0.006**
DBP [mmHg]	80.0 (70.0–85.0)	71.0 (69.5–80.0)	**0.001**
Smoking [%]	NA	23.1%	-
Resistin [ng/mL]	48.3 (35.2–66.4)	35.2(26.1–54.4)	**0.003**
TNF-alpha [pg/mL]	4.4 (3.5–5.6)	3.0 (2.5–4.0)	**<0.001**
CRP [mg/dL]	0.2 (0.1–0.4)	0.1 (0.1–0.4)	**0.020**
PAI-1 [ng/mL]	92.4 (71.6–119.4)	113.1 (85.8–149.8)	**0.008**

CKD, chronic kidney disease; eGFR, estimated glomerular filtration rate; BMI, body mass index; HgbA1c, haemoglobin A1c; HOMA-IR, homeostasis model assessment of insulin resistance; SBP, systolic blood pressure; DBP, diastolic blood pressure; TNF-alpha, tumor necrosis factor-alpha; CRP, C-reactive protein; PAI-1, plasminogen activator inhibitor; NA—not available; *p*-values < 0.05 are marked in bold.

**Table 2:** Resistin concentrations in CKD patients and the control group according to the estimated cardiovascular risk.

Cardiovascular Risk	eGFR < 60 mL/min/1.73 m^2^ *n* = 99		eGFR ≥ 60 mL/min/1.73 m^2^ *n* = 43	
	*n*	Median (IQR)	*n*	Median (IQR)
Very high-risk	70	52.1 (36.9–74.2)	7	45.8 (30.8–73.4)
High-risk	29	43.6 (29.1–54.4)	19	37.1 (25.2–60.6)
Moderate-risk	-	-	14	36.4 (29.9–52.6)
Low-risk	-	-	3	22.4 (16.7–26.1)
		***p* = 0.014**		p_trend_ = 0.087

CKD, chronic kidney disease; *p*-values < 0.05 are marked in bold.

**Table 3:** General Linear Model parameter estimates for the relationship between resistin concentration (ng/mL) and CV risk level, TNF-alpha and PAI-1 concentrations in CKD patients.

Variable	β Coefficient	95% CI for β	*p*-Value
CV risk			
very high	36.38	4.53;68.24	**0.026**
high	Ref.	-	-
TNF-alpha	−0.02	−2.67;2.63	0.989
TNF-alpha*very high CV risk	1.02	−1.83;3.87	0.478
TNF-alpha*high CV risk	Ref.	-	-
PAI-1	0.30	0.07;0.53	**0.012**
PAI-1*very high CV risk	−0.27	−0.54;−0.003	**0.048**
PAI-1*high CV risk	Ref.	-	-

CV risk, cardiovascular risk; TNF-alpha, tumor necrosis factor-alpha; PAI-1, plasminogen activator inhibitor; *p*-values < 0.05 are marked in bold.

## Data Availability

All relevant data analyzed during the current study are included in the article. Access to raw datasets may be provided upon reasonable request to the corresponding author following permission by the Ethics Committee and the Institute at which the study was conducted.

## Supplementary Materials

The following supporting information can be downloaded at: https://www.mdpi.com/article/10.3390/cells12070999/s1, Figure S1: Scenario 1 version of the Figure 1 from the main body of the manuscript; Figure S2: Scenario 2 version of the Figure 1 from the main body of the manuscript; Table S1: Scenario 1 version of the Table 2 from the main body of the manuscript; Table S2: Scenario 1 version of the Table 3 from the main body of the manuscript; Table S3: Scenario 2 version of the Table 2 from the main body of the manuscript; Table S4: Scenario 2 version of the Table 3 from the main body of the manuscript.

## References

[1] Holcomb I.N., Kabakoff R.C., Chan B., Baker T.W., Gurney A., Henzel W., Nelson C., Lowman H.B., Wright B.D., Skelton N.J. (2000). FIZZ1, a novel cysteine-rich secreted protein associated with pulmonary inflammation, defines a new gene family. EMBO J..

[2] Acquarone E., Monacelli F., Borghi R., Nencioni A., Odetti P. (2019). Resistin: A reappraisal. Mech. Ageing Dev..

[3] Steppan C.M., Bailey S.T., Bhat S., Brown E.J., Banerjee R.R., Wright C.M., Patel H.R., Ahima R.S., Lazar M.A. (2001). The hormone resistin links obesity to diabetes. Nature.

[4] Axelsson J., Bergsten A., Qureshi A.R., Heimbürger O., Bárány P., Lönnqvist F., Lindholm B., Nordfors L., Alvestrand A., Stenvinkel P. (2006). Elevated resistin levels in chronic kidney disease are associated with decreased glomerular filtration rate and inflammation, but not with insulin resistance. Kidney Int..

[5] Tarkowski A., Bjersing J., Shestakov A., Bokarewa M.I. (2010). Resistin competes with lipopolysaccharide for binding to toll-like receptor 4. J. Cell. Mol. Med..

[6] Lee S., Lee H.-C., Kwon Y.-W., Lee S.E., Cho Y., Kim J., Lee S., Kim J.-Y., Lee J., Yang H.-M. (2014). Adenylyl Cyclase-Associated Protein 1 Is a Receptor for Human Resistin and Mediates Inflammatory Actions of Human Monocytes. Cell Metab..

[7] Wolf G. (2004). Insulin Resistance and Obesity: Resistin, A Hormone Secreted by Adipose Tissue. Nutr. Rev..

[8] Filková M., Haluzík M., Gay S., Šenolt L. (2009). The role of resistin as a regulator of inflammation: Implications for various human pathologies. Clin. Immunol..

[9] Koch A., Gressner O.A., Sanson E., Tacke F., Trautwein C. (2009). Serum resistin levels in critically ill patients are associated with inflammation, organ dysfunction and metabolism and may predict survival of non-septic patients. Crit. Care.

[10] Schäffler A., Hamer O., Dickopf J., Goetz A., Landfried K., Voelk M., Herfarth H., Kopp A., Büchler C., Schölmerich J. (2010). Admission Resistin Levels Predict Peripancreatic Necrosis and Clinical Severity in Acute Pancreatitis. Am. J. Gastroenterol..

[11] Hutcheson J., Ye Y., Han J., Arriens C., Saxena R., Li Q.-Z., Mohan C., Wu T. (2015). Resistin as a potential marker of renal disease in lupus nephritis. Clin. Exp. Immunol..

[12] Degawa-Yamauchi M., Bovenkerk J.E., Juliar B.E., Watson W., Kerr K., Jones R., Zhu Q., Considine R.V. (2003). Serum Resistin (FIZZ3) Protein Is Increased in Obese Humans. J. Clin. Endocrinol. Metab..

[13] Fang W.Q., Zhang Q., Peng Y.B., Chen M., Lin X.P., Wu J.H., Cai C.H., Mei Y.F., Jin H. (2011). Resistin levelispositively correlated with thrombotic complications in Southern Chinese metabolic syndrome patients. J. Endocrinol. Investig..

[14] Wang L.-K., Wang H., Wu X.-L., Shi L., Yang R.-M., Wang Y.-C. (2020). Relationships among resistin, adiponectin, and leptin and microvascular complications in patients with type 2 diabetes mellitus. J. Int. Med. Res..

[15] Zhang Y., Li Y., Yu L., Zhou L. (2017). Association between serum resistin concentration and hypertension: A systematic review and meta-analysis. Oncotarget.

[16] Xu W., Yu L., Zhou W., Luo M. (2006). Resistin increases lipid accumulation and CD36 expression in human macrophages. Biochem. Biophys. Res. Commun..

[17] Calabrò P., Cirillo P., Limongelli G., Maddaloni V., Riegler L., Palmieri R., Pacileo G., De Rosa S., Pacileo M., De Palma R. (2011). Tissue Factor Is Induced by Resistin in Human Coronary Artery Endothelial Cells by the NF-ĸB-Dependent Pathway. J. Vasc. Res..

[18] Mu H., Ohashi R., Yan S., Chai H., Yang H., Lin P., Yao Q., Chen C. (2006). Adipokine resistin promotes in vitro angiogenesis of human endothelial cells. Cardiovasc. Res..

[19] Chen C., Jiang J., Lu J.-M., Chai H., Wang X., Lin P.H., Yao Q. (2010). Resistin decreases expression of endothelial nitric oxide synthase through oxidative stress in human coronary artery endothelial cells. Am. J. Physiol.-Heart Circ. Physiol..

[20] Jiang C., Zhang H., Zhang W., Kong W., Zhu Y., Zhang H., Xu Q., Li Y., Wang X. (2009). Homocysteine promotes vascular smooth muscle cell migration by induction of the adipokine resistin. Am. J. Physiol.-Cell Physiol..

[21] Zhou L., Li J.-Y., He P.-P., Yu X.-H., Tang C.-K. (2021). Resistin: Potential biomarker and therapeutic target in atherosclerosis. Clin. Chim. Acta.

[22] Weikert C., Westphal S., Berger K., Dierkes J., Möhlig M., Spranger J., Rimm E.B., Willich S.N., Boeing H., Pischon T. (2008). Plasma Resistin Levels and Risk of Myocardial Infarction and Ischemic Stroke. J. Clin. Endocrinol. Metab..

[23] Chemaly E.R., Hadri L., Zhang S., Kim M., Kohlbrenner E., Sheng J., Liang L., Chen J., K-Raman P., Hajjar R.J. (2011). Long-term in vivo resistin overexpression induces myocardial dysfunction and remodeling in rats. J. Mol. Cell. Cardiol..

[24] Frankel D.S., Vasan R.S., D’Agostino R.B., Benjamin E.J., Levy D., Wang T.J., Meigs J.B. (2009). Resistin, Adiponectin, and Risk of Heart Failure: The Framingham Offspring Study. J. Am. Coll. Cardiol..

[25] Hay S.I., Jayaraman S.P., Truelsen T., Sorensen R.J.D., Millear A., Giussani G., Beghi E. (2016). Global, regional, and national incidence, prevalence, and years lived with disability for 310 diseases and injuries, 1990–2015: A systematic analysis for the Global Burden of Disease Study 2015. Lancet.

[26] Mafham M., Emberson J., Landray M.J., Wen C.-P., Baigent C. (2011). Estimated Glomerular Filtration Rate and the Risk of Major Vascular Events and All-Cause Mortality: A Meta-Analysis. PLoS ONE.

[27] Ortiz P.A., Covic A., Fliser D., Fouque D., Goldsmith D., Kanbay M., Mallamaci F., Massy Z.A., Rossignol P., Vanholder R. (2014). Epidemiology, contributors to, and clinical trials of mortality risk in chronic kidney failure. Lancet.

[28] Cebeci E., Cakan C., Gursu M., Uzun S., Karadag S., Koldas M., Calhan T., Helvaci S.A., Ozturk S. (2019). The Main Determinants of Serum Resistin Level in Type 2 Diabetic Patients are Renal Function and Inflammation not Presence of Microvascular Complication, Obesity and Insulin Resistance. Exp. Clin. Endocrinol. Diabetes.

[29] Dan S., Aditya P., Banerjee P., Bal C., Roy H., Banerjee I. (2014). Effect of chronic kidney disease on serum resistin level. Niger. J. Clin. Pract..

[30] Liu G., Deng Y., Sun L., Ye X., Yao P., Hu Y., Wang F., Ma Y., Li H., Liu Y. (2016). Elevated plasma tumor necrosis factor-α receptor 2 and resistin are associated with increased incidence of kidney function decline in Chinese adults. Endocrine.

[31] Munjas J., Sopić M., Bogavac-Stanojević N., Kravljača M., Miljković M., Simić-Ogrizović S., Spasojević-Kalimanovska V., Jelić-Ivanović Z. (2020). Serum Resistin, Adenylate Cyclase-Associated Protein 1 Gene Expression, and Carotid Intima-Media Thickness in Patients with End-Stage Renal Disease and Healthy Controls. Cardiorenal. Med..

[32] Marouga A., Dalamaga M., Kastania A.N., Kroupis C., Lagiou M., Saounatsou K., Dimas K., Vlahakos D.V. (2016). Circulating resistin is a significant predictor of mortality independently from cardiovascular comorbidities in elderly, non-diabetic subjects with chronic kidney disease. Biomarkers.

[33] Koppe L., Fouque D., Kalantar-Zadeh K. (2019). Kidney cachexia or protein-energy wasting in chronic kidney disease: Facts and numbers. J. Cachexia Sarcopenia Muscle.

[34] Marouga A., Dalamaga M., Kastania A.N., Antonakos G., Thrasyvoulides A., Kontelia G., Dimas C., Vlahakos D.V. (2013). Correlates of serum resistin in elderly, non-diabetic patients with chronic kidney disease. Clin. Lab..

[35] Visseren F.L.J., Mach F., Smulders Y.M., Carballo D., Koskinas K.C., Bäck M., Benetos A., Biffi A., Boavida J.-M., Capodanno D. (2021). ESC Guidelines on cardiovascular disease prevention in clinical practice. Eur. Heart J..

[36] Piepoli M.F., Hoes A.W., Agewall S., Albus C., Brotons C., Catapano A.L., Cooney M.-T., Corrà U., Cosyns B., Deaton C. (2016). 2016 European Guidelines on cardiovascular disease prevention in clinical practice: The Sixth Joint Task Force of the European Society of Cardiology and Other Societies on Cardiovascular Disease Prevention in Clinical Practice (constituted by representatives of 10 societies and by invited experts). Developed with the special contribution of the European Association for Cardiovascular Prevention & Rehabilitation (EACPR). Eur. Heart J..

[37] Rae C., Robertson S.A., Taylor J.M.W., Graham A. (2007). Resistin induces lipolysis and re-esterification of triacylglycerol stores, and increases cholesteryl ester deposition, in human macrophages. FEBS Lett..

[38] Kawanami D., Maemura K., Takeda N., Harada T., Nojiri T., Imai Y., Manabe I., Utsunomiya K., Nagai R. (2004). Direct reciprocal effects of resistin and adiponectin on vascular endothelial cells: A new insight into adipocytokine–endothelial cell interactions. Biochem. Biophys. Res. Commun..

[39] Asgary S., Samsamshariat S.Z.A., Sakhaei F., Salehizadeh L., Keshvari M. (2019). Relationship between Resistin, Endothelin-1, and Flow-Mediated Dilation in Patient with and without Metabolic Syndrome. Adv. Biomed. Res..

[40] Hsu W.-Y., Chao Y.-W., Tsai Y.-L., Lien C.-C., Chang C.-F., Deng M.-C., Ho L.-T., Kwok C.F., Juan C.-C. (2011). Resistin induces monocyte-endothelial cell adhesion by increasing ICAM-1 and VCAM-1 expression in endothelial cells via p38MAPK-dependent pathway. J. Cell. Physiol..

[41] Subodh V., Shu-Hong L., Chao-Hung W., Paul W.M., Ren-Ke L., Richard D.W., Donald A.G. (2003). Resistin Promotes Endothelial Cell Activation. Circulation.

[42] Tisato V., Romani A., Tavanti E., Melloni E., Milani D., Bonaccorsi G., Sanz J.M., Gemmati D., Passaro A., Cervellati C. (2019). Crosstalk Between Adipokines and Paraoxonase 1: A New Potential Axis Linking Oxidative Stress and Inflammation. Antioxidants.

[43] Melone M., Wilsie L., Palyha O., Strack A., Rashid S. (2012). Discovery of a New Role of Human Resistin in Hepatocyte Low-Density Lipoprotein Receptor Suppression Mediated in Part by Proprotein Convertase Subtilisin/Kexin Type 9. J. Am. Coll. Cardiol..

[44] Jurin I., Paić F., Bulimbašić S., Rudež I., Đerek L., Jurin H., Knežević A., Starcevic B., Ajduk M. (2018). Association between Circulatory and Plaque Resistin Levels with Carotid Plaque Instability and Ischemic Stroke Events. Heart Surg. Forum.

[45] Costandi J., Melone M., Zhao A., Rashid S. (2011). Human Resistin Stimulates Hepatic Overproduction of Atherogenic ApoB-Containing Lipoprotein Particles by Enhancing ApoB Stability and Impairing Intracellular Insulin Signaling. Circ. Res..

[46] Norman G., Woodiwiss A.J., Peterson V., Gomes M., Sareli P., Norton G.R. (2020). Impact of metabolic and inflammatory changes on glomerular function beyond conventional risk factors in an urban South Africa community with prevalent obesity. Cardiovasc. J. Afr..

[47] Karbach S., Wenzel P., Waisman A., Munzel T., Daiber A. (2014). eNOS Uncoupling in Cardiovascular Diseases—The Role of Oxidative Stress and Inflammation. Curr. Pharm. Des..

[48] Harrison D.G., Guzik T.J., Lob H.E., Madhur M.S., Marvar P.J., Thabet S.R., Vinh A., Weyand C.M. (2011). Inflammation, Immunity, and Hypertension. Hypertension.

[49] Ohara Y., Peterson T.E., Harrison D.G. (1993). Hypercholesterolemia increases endothelial superoxide anion production. J. Clin. Investig..

[50] Hanna R.M., Ghobry L., Wassef O., Rhee C.M., Kalantar-Zadeh K. (2020). A Practical Approach to Nutrition, Protein-Energy Wasting, Sarcopenia, and Cachexia in Patients with Chronic Kidney Disease. Blood Purif..

[51] Ridker P.M., MacFadyen J.G., Glynn R.J., Koenig W., Libby P., Everett B.M., Lefkowitz M., Thuren T., Cornel J.H. (2018). Inhibition of Interleukin-1β by Canakinumab and Cardiovascular Outcomes in Patients with Chronic Kidney Disease. J. Am. Coll. Cardiol..

[52] Cobo G., Lindholm B., Stenvinkel P. (2018). Chronic inflammation in end-stage renal disease and dialysis. Nephrol. Dial. Transplant..

[53] Yaturu S., Reddy R.D., Rains J., Jain S.K. (2007). Plasma and urine levels of resistin and adiponectin in chronic kidney disease. Cytokine.

[54] Ding Y., Li X. (2019). Resistin Promotes Thrombosis in Rats with Deep Vein Thrombosis via Up-Regulating MMP-2, MMP-9, and PAI-1. Clin. Lab..

[55] Ikeda Y., Tsuchiya H., Hama S., Kajimoto K., Kogure K. (2014). Resistin regulates the expression of plasminogen activator inhibitor-1 in 3T3-L1 adipocytes. Biochem. Biophys. Res. Commun..

[56] Qi Q., Wang J., Li H., Yu Z., Ye X., Hu F.B., Franco O.H., Pan A., Liu Y., Lin X. (2008). Associations of resistin with inflammatory and fibrinolytic markers, insulin resistance, and metabolic syndrome in middle-aged and older Chinese. Eur. J. Endocrinol..

[57] Beier J.I., Guo L., von Montfort C., Kaiser J.P., Joshi-Barve S., Arteel G.E. (2008). New Role of Resistin in Lipopolysaccharide-Induced Liver Damage in Mice. Experiment.

[58] Lijnen H.R., Bachmann F., Collen D., Ellis V., Pannekoek H., Rijken D.C., Thorsen S. (1994). Mechanisms of plasminogen activation. J. Intern. Med..

[59] Collen D., Lijnen H., Plow E.F. (1986). The fibrinolytic system in man. Crit. Rev. Oncol..

[60] Sprengers E.D., Kluft C. (1987). Plasminogen activator inhibitors. Blood.

[61] Kohler H.P., Grant P.J. (2000). Plasminogen-Activator Inhibitor Type 1 and Coronary Artery Disease. N. Engl. J. Med..

[62] Thögersen A.M., Jansson J.-H., Boman K., Nilsson T.K., Weinehall L., Huhtasaari F., Hallmans G. (1998). High Plasminogen Activator Inhibitor and Tissue Plasminogen Activator Levels in Plasma Precede a First Acute Myocardial Infarction in Both Men and Women: Evidence for the fibrinolytic system as an independent primary risk factor. Circulation.

[63] Christ G., Nikfardjam M., Huber-Beckmann R., Gottsauner-Wolf M., Glogar D., Binder B.R., Wojta J., Huber K. (2005). Predictive value of plasma plasminogen activator inhibitor-1 for coronary restenosis: Dependence on stent implantation and antithrombotic medication. J. Thromb. Haemost..

[64] Alessi M.-C., Nicaud V., Scroyen I., Lange C., Saut N., Fumeron F., Marre M., Lantieri O., Fontaine-Bisson B., Juhan-Vague I. (2011). Association of vitronectin and plasminogen activator inhibitor-1 levels with the risk of metabolic syndrome and type 2 diabetes mellitus. Thromb. Haemost..

[65] Van De Craen B., Declerck P.J., Gils A. (2012). The Biochemistry, Physiology and Pathological roles of PAI-1 and the requirements for PAI-1 inhibition in vivo. Thromb. Res..

[66] Shirakawa J., Togashi Y., Tajima K., Orime K., Kikuchi K., Miyazaki T., Sato K., Kimura M., Goshima Y., Terauchi Y. (2012). Plasminogen activator inhibitor-1 is associated with renal dysfunction independent of BMI and serum lipid levels in patients with type 2 diabetes. Diabetes Res. Clin. Pract..

[67] Owecki M., Miczke A., Nikisch E., Pupek-Musialik D., Sowiński J. (2011). Serum Resistin Concentrations are Higher in Human Obesity but Independent from Insulin Resistance. Exp. Clin. Endocrinol. Diabetes.

[68] Hasegawa G., Ohta M., Ichida Y., Obayashi H., Shigeta M., Yamasaki M., Fukui M., Yoshikawa T., Nakamura N. (2005). Increased serum resistin levels in patients with type 2 diabetes are not linked with markers of insulin resistance and adiposity. Acta Diabetol..

[69] Lee J.H., Chan J.L., Yiannakouris N., Kontogianni M., Estrada E., Seip R., Orlova C., Mantzoros C.S. (2003). Circulating Resistin Levels Are Not Associated with Obesity or Insulin Resistance in Humans and Are Not Regulated by Fasting or Leptin Administration: Cross-Sectional and Interventional Studies in Normal, Insulin-Resistant, and Diabetic Subjects. J. Clin. Endocrinol. Metab..

