# Supplementary material for: Resistin Contribution to Cardiovascular Risk in Chronic Kidney Disease Male Patients
Source: Cells. 2023 Mar 24;12(7):999. doi: 10.3390/cells12070999 (PMC10093733; doi:10.3390/cells12070999)
Supplement: Supplementary file 1 [file cells-12-00999-s001.zip › cells-2257661-supplementary final .pdf]

Supplementary file

Scenario 1.All patients with missing data on smoking status are coded as non-smokers

Table S1. Scenario 1 version of the Table 2 from the main body of the manuscript.

Cardiovascular risk	Study group n=99		Control group n=43		p-value
	n	Median (IQR)	n	Median (IQR)	
Very high-risk	49	52.7 (44.0-75.5)	5	45.8 (32.1-73.2)	0.335
High-risk	51	43.6 (30.2-58.0)	21	37.1 (27.1-61.0)	0.545
Moderate-risk	-	-	13	36.4 (29.9-52.6)	-
Low-risk	-	-	3	22.4 (16.7-26.1)	-
		**p=0.003**		p_{trend}=0.102	

Residual analysis for CKD patients: significant association between resistin residuals and PAI-1

Table S2. Scenario 1 version of the Table 3 from the main body of the manuscript.

Variable	β coefficient	95% CI for β	p-value
CV risk			
very high	40.63	14.03;67.23	**0.003**
high	Ref.	-	-
PAI-1	0.26	0.07;0.46	**0.009**
PAI-1*very high CV risk	-0.26	-0.51;-0.01	**0.039**
PAI-1*high CV risk	Ref.	-	-

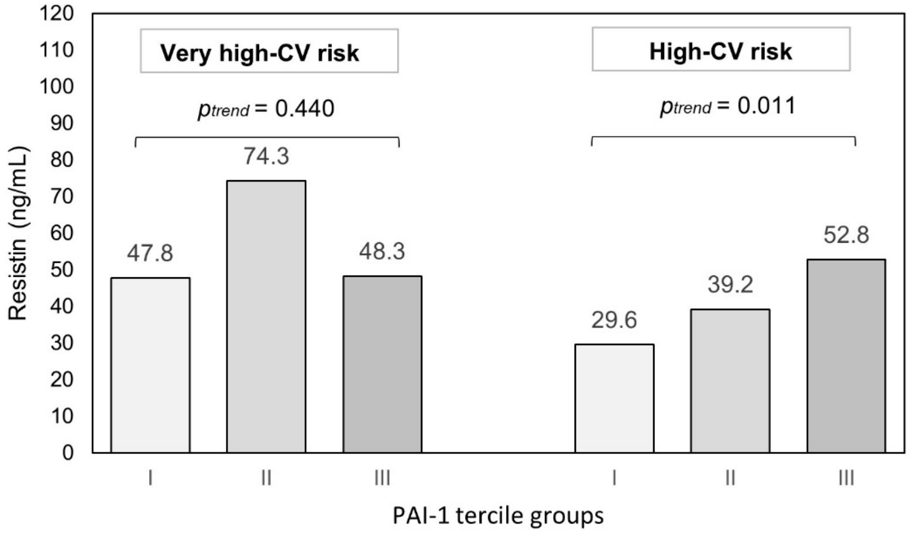

Figure S1. Scenario 1 version of the Figure 1 from the main body of the manuscript.

Scenario 2.All patients with missing data on smoking status are coded as smokers

Table S3. Scenario 2 version of the Table 2 from the main body of the manuscript.

Cardiovascular risk	Study group n=99		Control group n=43		p-value
	n	Median (IQR)	n	Median (IQR)	
Very high-risk	82	50.5 (36.2-71.5)	8	39.5 (29.6-67.3)	0.263
High-risk	17	44.7 (28.0-53.4)	18	39.6 (28.0-60.8)	0.935
Moderate-risk	-	-	13	36.4 (29.9-52.6)	-
Low-risk	-	-	3	22.4 (16.7-26.1)	-
		p=0.073		p_{trend}=0.152	

Residual analysis for CKD patients: significant association between resistin residuals and TNF-alpha and PAI-1 (at the border of significance)

Table S4. Scenario 2 version of the Table 3 from the main body of the manuscript.

Variable	β coefficient	95% CI for β	p-value
CV risk			
very high	35.73	-14.32;85.79	0.160
high	Ref.	-	-
TNF-alpha	-0.85	-7.48;5.78	0.800
TNF-alpha*very high CV risk	1.83	-4.87;8.54	0.588
TNF-alpha*high CV risk	Ref.	-	-
PAI-1	0.38	-0.003;0.76	0.052
PAI-1*very high CV risk	-0.33	-0.73;0.07	0.107
PAI-1*high CV risk	Ref.	-	-

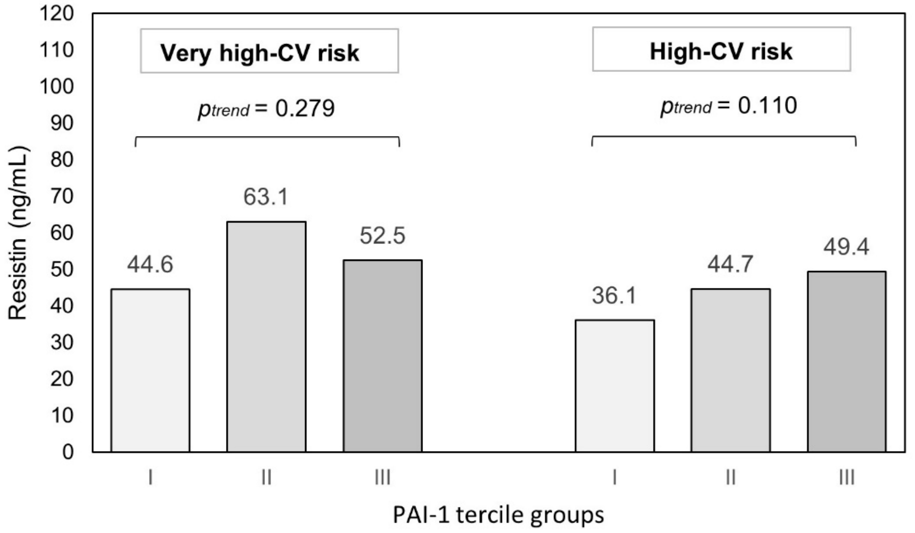

Figure S2. Scenario 2 version of the Figure 1 from the main body of the manuscript.
